# Oral Hygiene and Care of the Teeth

**Published:** 1887-04

**Authors:** C. E. H. Phillips

**Affiliations:** New York


### ORAL HYGIENE AND CARE OF THE TEETH.

BY C. E. H. PHILLIPS, D. D. S., NEW YORK.

There is nothing more essential to the preservation of the teeth and the normal standard of their surrounding tissues than the judicious use of the brush. Most persons of education and refinement cleanse the teeth at least once a day, and that generally at the time of their morning ablution; a smaller number also use the brush regularly before retiring, and there are those who do so after each meal; but of all these, the proportion of those who use the brush properly is wofully small. This is evidenced by the teeth and gums as they are presented to the dental practitioner, and by the exclamations of surprise on the part of the patient upon being instructed in this particular.

It is, of course, desirable that the teeth should be brushed upon arising in the morning, and the mouth rinsed after each meal sufficiently to remove food fragments, but it is of infinitely greater importance that they should be thoroughly cleansed before retiring at night, that all the interstices and irregularities between the teeth may be freed from particles of food, which would otherwise be mixed with the saliva, and if allowed to remain in the mouth during the long hours of repose become acidulated, ferment and decompose, causing incipient decay and hastening it where already commenced. The brush, then, is the all-important factor in the maintenance of a cleanly mouth, and in the selection of it patients are oftener puzzled. Nor is this to be wondered at, when every druggist and many venders of fancy wares offer various sizes and designs, the most of them ill adapted to their special requirements, and perhaps causing positive injury when an improper choice is made. When doubt arises concerning selection the dentist should be consulted.

In the majority of cases a small brush will accomplish a better purpose than a large one, from its ease of application, both inside and outside the dental arches. It should be borne in mind that, with use, a new brush soon softens; therefore, when selected, the bristles should be fairly stiff, not too closely placed, and if corrugated they will be found more advantageous in displacing particles between the teeth. In the use of the brush, too, many are content with the energetic rubbing of the same across the faces of the teeth, often to the injury of the gums. This is a mistake, for it should not be a severe operation, but a delicate one, to be carefully performed.

After brushing with a semi-rotary movement the free surfaces of the teeth, distributing the attention equally to those well back in the arch, the brush should be patted upon and against the gums and teeth at their necks, and passed (by a slight turning of the hand) in the direction of the length of the teeth, toward their cutting edges. This brushing from above downward in the upper arch, and the reverse in the lower, stimulates the gums and induces them to cling to the necks of the teeth, instead of forcing them back, which may be done by too harsh brushing across them. The bristles are forced between the teeth, freeing their approximal edges of accumulations.

Though more difficult of access, the inside surfaces should in no wise be neglected. The unfortunate, irregularly crowded position of teeth in the mouths of many individuals, renders it imperative that something more searching than the brush be employed, the bristles not completely passing between them. In such cases the judicious employment, after meals, of a tooth-pick, either quill or wood, and the frequent use of waxed dental floss, to which a little powder may be attached, will prove a great benefit.

Water alone is not sufficient to free the teeth from mucous secretions, hence the question naturally follows: With what should the teeth be cleansed? A good powder is of great assistance. As with brushes, so with powders, pastes and washes; their number is legion, of every variety and of different flavors. Though excellent preparations are offered, many are manufactured with little or no knowledge of the essential requirements. The dentrifice should be carefully selected, of the finest quality and easily removed from the free margins of the gums by rinsing. A little white Castile soap may be used, either with or without the powder, with cleansing and beneficial effect. Charcoal should not be used in any form. A simple, though grateful and very efficacious mouth-wash for soft and spongy gums, is common salt and water.

Children should be instructed in the habit of brushing the teeth at as early an age as possible.

When patients appreciate the importance of oral hygiene and practice cleanliness in the mouth, with the consequent maintenance of the normal condition of the secretions, much pain will be avoided, many teeth saved and a decrease in such diseases as pyorrhœa alveolaris and pericementitis must result.

The following excellent general directions for the care of the teeth have been issued by the Medical Committee of the National Dental Hospital, London:

1. The teeth should be cleaned at least once a day, the best time being at night—the last thing. For this purpose use a soft brush, on which take a little soap and then some prepared chalk, brushing up and down and across. There is rarely any objection to the friction causing the gum to bleed slightly.

2. Avoid all rough usage of the teeth, such as cracking nuts, biting thread, etc., as by so doing even good sound teeth may be injured.

3. When decay is first observed advice should at once be sought. It is the stopping in a small hole that is of the greatest service, though not infrequently a large filling preserves the tooth for years.

4. It is of the greatest importance that children, from four years and upward, should have their teeth frequently examined by the dental surgeon to see that the first set, particularly the back teeth, are not decaying too early, and to have the opportunity of timely treatment for the regulation and preservation of the second set.

5. Children should be taught to rinse the mouth night and morning, and to begin the use of the brush early; likewise the tooth-pick.

6. With regard to the food of children, to those who are old enough, whole-meal bread, porridge and milk should be given. This is much more wholesome and substantial food than white bread.

7. If the foregoing instructions were carried out, comparatively few teeth would have to be extracted.

